# Template-Free Synthesis of N-Doped Porous Carbon Materials From Furfuryl Amine-Based Protic Salts

**DOI:** 10.3389/fchem.2020.00196

**Published:** 2020-03-31

**Authors:** Yan Zhang, Jixia Wang, Guohong Shen, Junfei Duan, Shiguo Zhang

**Affiliations:** ^1^College of Materials Science and Engineering, Hunan Province Key Laboratory for Advanced Carbon Materials and Applied Technology, Hunan University, Changsha, China; ^2^School of Materials Science and Engineering, Changsha University of Science and Technology, Changsha, China

**Keywords:** template-free, nitrogen-doped porous carbon, furfuryl amine, protic salts, anions

## Abstract

Nitrogen-doped porous carbon materials (NPCMs) are usually obtained by carbonization of complicated nitrogen-containing polymers in the presence of template or physical/chemical activation of the as-synthesized carbon materials. Herein we reported the facile synthesis of NPCMs by direct carbonization of a series of furfuryl amine (FA)-based protic salts ([FA][X], X = NTf_2_, HSO_4_, H_2_PO_4_, CF_3_SO_3_, BF_4_, NO_3_, Cl) without any templates, tedious synthetic steps and other advanced techniques. The thermal decomposition of precursors and structure, elemental composition, surface atomic configuration, and porosity of carbons have been carefully investigated by thermogravimetric analysis (TGA), X-ray diffraction (XRD), Raman spectra, X-ray photoelectron spectroscopy (XPS), combustion elemental analysis, energy-dispersive spectrometry, and nitrogen isotherm adsorption. Different from the parent amine FA that was evaporated below 130°C and no carbon was finally obtained, it was found that all the prepared protic precursors yield NPCMs. These carbon materials were found to exhibit anion structure- dependent carbon yield, chemical composition, and porous structure. The obtained NPCMs can be further exploited as adsorbents for dye removal and decoloration. Among all NPCMs, [FA][H_2_PO_4_]-derived carbon owing to its high surface area and special pore structure exhibits the highest adsorption capacities toward both Methylene blue and Rhodamine B.

## Introduction

Porous carbons have attracted great interest, due to their potential applications in diverse areas including environmental treatment, sensor, catalysis, energy conversion, and storage, etc (Nishihara and Kyotani, 2012; Fang et al., 2013; Su et al., 2013). Particularly, porous carbon materials have been recently used for harvesting water energy into electric energy based on new phenomena of “hydrovoltaic effect” (Tang and Yang, 2016; Xue et al., 2017; Zhang et al., 2018; Han et al., 2019). In order to develop the porous network in carbon materials, the application of hard or soft template method is often employed (Kyotani, 2006; Yamauchi and Kuroda, 2008; Xia et al., 2010; Ma et al., 2013; Petkovich and Stein, 2013). The hard-templating method requires tedious procedures including synthesis and combination of precursor and template, polymerization, prolysis, and removal of template via caustic chemicals (Ryoo et al., 1999; Jun et al., 2000; Joo et al., 2001), while the soft templating method often involves toxic agents, special membrane morphologies, time-consuming polymerization and solvent evaporation-induced self-assembly process (Liang et al., 2004; Tanaka et al., 2005; Zhang et al., 2005; Liu et al., 2013). Porous carbons can also be obtained through physical (O_2_, CO_2_) or chemical (KOH, H_3_PO_4_, ZnCl_2_) activation (Teng and Wang, 2000; Puziy et al., 2002a,b; Xia et al., 2007; Wang and Kaskel, 2012). The harsh activation methods usually need additional carbonization step and are uncontrollable in porosity. In contrast to the time-consuming templating or activation methods, direct synthesis of porous carbons from simple precursors via template-free carbonization is much more feasible and highly attractive. Indeed, there are several works related to direct synthesis from precursors such as metal organic frameworks (MOFs) (Shen and Bai, 2010; Hu et al., 2012), organic metal salts (Maruyama et al., 2010; Sevilla and Fuertes, 2014), crude plant materials (Titirici et al., 2007), self-assembled block copolymers (Zhong et al., 2012), hydrogen-bonding supramolecular precursors (Zhang et al., 2015e), Nevertheless, these methods appear to be challenging and still suffer from detrimental issues such as less developed porosity, absence of heteroatom doping, uncontrolled structure and properties, and complicated synthesis.

Other than the construction of porous structure, modification of carbon structure with heteroatoms was shown to modulate their physicochemical properties and produce functional groups on the carbon architecture (Su et al., 2011). For example, N doping of carbon materials was reported to modify the conductivity, basicity, oxidation-resistance ability, and catalytic performance (Yang et al., 2005; Shao et al., 2008; Gong et al., 2009; Zhang et al., 2014a,b). Heteroatom-doping can be generally achieved either by post-modification or by in situ carbonization. Post-modification usually needs heteroatom-containing chemicals and the content and distribution of doping elements cannot be easily controlled (Choi et al., 2012; Zheng et al., 2012). Contrastively, in situ synthesis from specific precursors is liable to produce carbon materials with uniformly distributed dopants (Shao et al., 2008).

Recently, ionic liquids (ILs) were demonstrated to be potential precursors of N-doped carbons due to their negligible volatility, intrinsic N-containing nature, and tunable structure. Such attractive properties simplifies the carbonization process drastically and renders the resulted carbon materials with high N content in homogeneous state and high conductivity for potential applications (Lee et al., 2009, 2010; Paraknowitsch et al., 2010; Wang and Dai, 2010; Yang et al., 2011; Fellinger et al., 2012, 2013; Fechler et al., 2013; Sahraie et al., 2014; Xie and Su, 2015; Zhang et al., 2015a; Zhu et al., 2015). Unfortunately, only very few specific aprotic ILs containing cyano/nitrile moieties can substantially produce carbon and the large scale production of carbon from these high-cost ILs are blocked. Recently, we reported that protic ILs and salts (PILs/PSs) can be employed as versatile precursors for the direct synthesis of N-doped carbons (Zhang et al., 2014a). In contrast to the reported aprotic ILs, PILs/PSs could be easily obtained by simple neutralization of basic amines with inorganic/organic acids in very short time without further purification. PILs/PSs could yield NPCMs whose structures and properties depending strongly on the nature of precursors. Furthermore, the correlations between protic precursors and carbon materials, especially the effect of the cationic structure (amine) of PILs/PSs on the structure and properties of the obtained carbons, such as carbon yield, graphitic degree, nitrogen content, and porous structure, have been carefully studied and established (Zhang et al., 2014a,b, 2015d,e,f). A detrimental point is that only PILs/PSs containing very few kinds of anions such as [HSO_4_] and [CF_3_SO_3_] could give rise to substantial carbon residues (Zhang et al., 2014a,b), while most other anions failed to ensure successful carbonization because of the strong nucleophilicities and thermal instabilities, and the influence of anions (acids) on the structure and properties of the PILs/PSs-derived NPCMs is still unknown.

In this work, using furfuryl amine (FA) as a specific base, we reported the synthesis of nitrogen-doped porous carbon materials (NPCMs) by direct carbonization of a series of FA-based PSs with various anions ([FA][X], X = NTf_2_, HSO_4_, H_2_PO_4_, CF_3_SO_3_, BF_4_, NO_3_, Cl). Different from the results obtained from previous PILs/PSs, all [FA][X] salts being investigated were found to produce N-doped carbon materials. Depending on the anions employed, we can also co-dop N and other heteroatoms such as S, P, B into the carbon skeleton. The anions of the precursors obviously exert a large influence on the carbon yield, elemental composition, surface area, pore structure, and dye adsorption capabilities of the obtained NPCMs. Particularly, [FA][H_2_PO_4_]-derived carbon possess a very large specific surface area of up to 1380 m^2^/g, which is comparable to or even higher than that of most carbon materials generated by templating methods or activation. As a result, this highly porous carbon exhibits an excellent performance toward dye removal, with its adsorption capacity superior to some conventional adsorbents including reported porous carbon materials.

## Experimental Section

### Chemicals

Furfuryl amine (98%), sulfuric acid (95%), nitric acid (67%), fluoroboric acid (42%), and hydrochloric acid (35%-37%) were obtained from adamas-beta of Titan science and technology Co., Ltd. 1,1,1-trifluoro-N-[(trifluoromethyl)sulfonyl]methanesulfonamide (99%) and hexafluorophosphoric acid (60.0%) were obtained from KANTO chemical Co. Inc. Methylene blue (98%), Rhodamine B (95%), phosphoric acid (85%), and trifluoromethanesulfonic acid were purchased from TCI. An ultra-pure purification system (Master-S15Q, Hitech Instruments Co. Ltd., Shanghai, China) was used to produce 18.2 MΩ/cm water in all experiments.

### Synthesis of Protic Salts

All PSs were prepared by simple stoichiometric neutralization between FA and acids in an aqueous/organic solution, followed by solvent removal, according to the established procedure (Zhang et al., 2014a,b, 2015d,e).

### Preparation of N-Doped Porous Carbons

Protic salt [FA][X] was put into a boat inserted within an alumina tube placed in a horizontal tube furnace. The tube fitted with end caps was thoroughly flushed with inert gas (argon) for at least 30 min to remove air from the tube. Then the sample was heated at a rate of 10°C min^−1^ to 1,000°C and held at this temperature for 2 h under ambient pressure. After cooling to room temperature, the obtained product was ground into fine powders with an agate mortar and pestle for further characterization.

### Characterization

Thermogravimetric analysis (TGA) was conducted on a Seiko thermo-gravimetry/differential thermal analyzer (TG-DTA 6200) from room temperature to 1,000°C at a heating rate of 10°C min^−1^ under an N_2_ flow of 200 mL min^−1^, except for [FA][NO_3_] at a much smaller heating rate of 1°C min^−1^. The powder X-ray diffraction (XRD) patterns were collected on a Rigaku RINT-2000 diffractometer using Cu Ka (λ = 0.154 nm) radiation. The diffractograms were recorded in 2θ range from 10° to 90° with a 2θ step size of 0.02° and a scanning speed of 10° min^−1^. Nitrogen sorption isotherms were recorded on a JW-BK200C (Beijing JWGB SCI. & Tech. Co., Ltd) sorption analyzer at 77 K. All samples were purged with flowing N_2_ at 300°C for 3 h prior to measurement. The specific surface area (*S*_BET_) was calculated using the Brunauer–Emmett–Teller (BET) method from the nitrogen adsorption data in the range of relative pressure (p/p_0_) of 0.05–0.20. The total pore volume (*V*_tot_) was determined from the amount of N_2_ uptake at relative pressures (p/p_0_) of 0.99. The pore size distribution was calculated using the non-local density functional theory (NLDFT) method with the slit/cylinder pore model. The morphology was observed on JSM-7001F field emission scanning electron microscope (FE-SEM) using an accelerating voltage of 5.0 kV. Elemental composition analysis was conducted on JSM-7001F with an energy-dispersive spectrometry (EDS) accessory. Combustion analysis of carbon materials was performed with a vario-EL III CHN elemental analyzer to determine the content of nitrogen, carbon and hydrogen. Chemical state, compositions, and valence band spectra were analyzed by a PHI Quantera SXM X-ray photoelectron spectroscopy (XPS) with a base pressure of 6.7 × 10^−8^ Pa, with an Al Kα (1486.6 eV) as the X-ray source and a pass energy of 280.00 (survey scan) and 69.00 eV (high-resolution scan). The surface atomic composition of carbons was estimated by the analysis of the peak areas for each element by considering the atomic sensitivity of XPS. The high-resolution XPS spectra were analyzed by least-squares fitting analysis. UV–visible absorption spectroscopy was performed on SHIMADZU UV-2700 spectrophotometer using 1 mm cell.

### Dye Adsorption Experiments

Organic dyes discharged by the textile and tannery industries are primary pollutants of water sources. Sorption is known to be efficient way for dye removal and decoloration and a variety of materials, such as porous carbon (mainly activated carbon, carbon black, and activated carbon fiber), have been reported as promising adsorbents (Demirbas, 2009; Zhuang et al., 2009; Liang et al., 2012; Torad et al., 2014; Yagub et al., 2014). All adsorption kinetic experiments are performed at ambient temperature. Dye solutions of different concentrations were prepared by dissolving appropriate amounts of Methylene Blue (MB) or Rhodamine B (RhB) into deionized water. In a typical experiment of MB adsorption, 10 mg of the porous carbon was dispersed in 20 mL MB aqueous solution (80 mg L^−1^) under stirring in dark to ensure the establishment of an adsorption-desorption equilibrium. Then, aliquots (0.5 mL) of the samples at different time intervals were collected and filtered. The obtained transparent solution was then subjected to UV–vis absorption spectra analysis at 663 nm to monitor the adsorption process. The adsorption study of RhB was similar to MB except for the detection wavelength difference (554 nm). The concentration reserved in the adsorbent phase (*Q*_e_, mg/g) is measured using the following equation:

$$\begin{array}{l} Q_{e}=\frac{\left(C_{0}-C_{e}\right)V}{m} \end{array}$$

Where *C*_0_ (mg L^−1^) is the initial dye concentration, *C*_e_ (mg L^−1^) is the equilibrium concentration of dye in the aqueous solution, *V* (L) is the volume of solution and *m* (g) is the mass of the adsorbent. The adsorption isotherm was obtained by varying the initial dye concentration, which was then fitted by the Langmuir adsorption model:

$$\begin{array}{l} Q_{e}=\frac{Q_{m}bC_{e}}{1+bC_{e}} \end{array}$$

Where *Q*_e_ (mg g^−1^) is the amount of dyes adsorbed at equilibrium, *Q*_m_ (mg L^−1^) is the maximum adsorption capacity corresponding to complete monolayer coverage, *C*_e_ (mg L^−1^) is the equilibrium solute concentration and *b* (L mg ^−1^) is the equilibrium constant.

## Results and Discussion

### Thermal Decomposition of [FA][X]

**Scheme 1:**
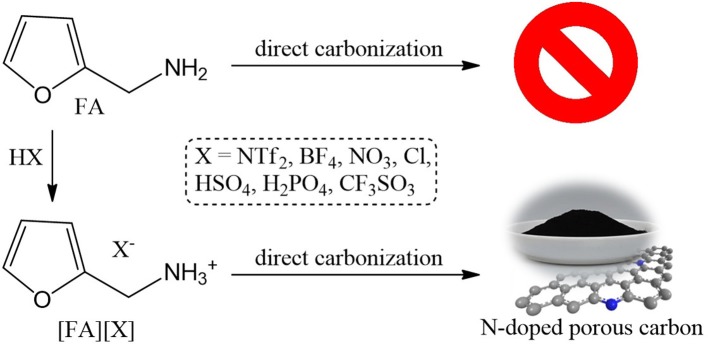
Synthesis of N-doped porous carbons by direct carbonization of [FA][X].

**Figure 1:**
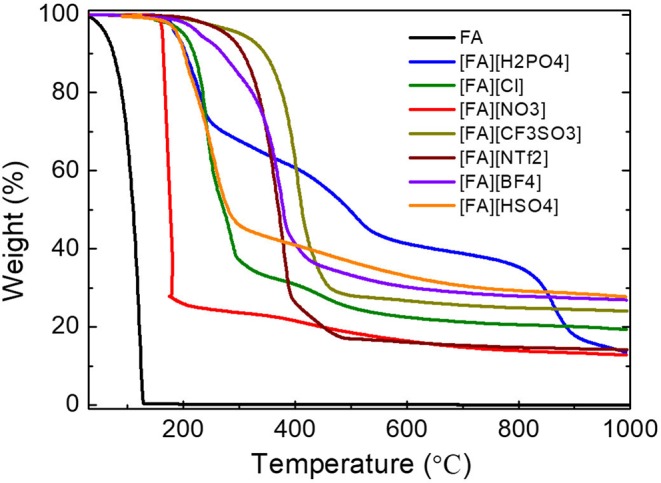
TGA curves of fa and its PSs pairing with various anions (heating rate: 10°C/min under argon flow 200 mL/min). Note that [FA][NO_3_] was heated at a heating rate of 1°C min^−1^.

All PSs precursors were synthesized by neutralization of FA with a series of Brønsted acids (Scheme 1). As shown in Table 1, among all of the obtained PSs, only [FA][NTf_2_] is liquid at room temperature, which can be denoted as protic IL. This is not unexpected because the [NTf_2_] anion which possesses low symmetry, bulky structure, and extensive electron delocalization always leads to the formation of ILs with relatively low melting point (Martinelli et al., 2011). Thermalgravimetric analysis (TGA) curves of [FA][X] are shown in Figure 1 along with FA for comparison. Obviously, the parent amine was completely evaporated below 130°C and no carbon was finally obtained, while all of the corresponding PSs paired with variable anions show higher thermal stability and indeed give rise to carbon residues. This result suggested the great potentials of using these PSs as new low-molecular-weight precursors. The successful carbonization is related strongly to the protonation that improved the thermal stability of PSs and facilitate the polymerization, condensation and fusion during complicated carbonization process (Zhang et al., 2014a,b).

**Table 1:** Characteristics of [FA][X]-derived NPCs.

**PSs**^**a**^	**State**^**a**^	**Oven yield (wt%)**^**b**^	**Relative yield (%)**^**c**^	***S*_**BET**_ (m^**2**^ g^**−1**^)**	***v*_**tot**_ (cm^**3**^ g^**−1**^)**	**N (wt%)**^**d**^	**C (wt%)**^**d**^	**H (wt%)**^**d**^	**N (at%)**^**e**^	**O (at%)**^**e**^	**X (at%)**^**e**^
[FA][NTf_2_]	L	14.6 (22.2)	65.8	657	0.509	3.241	82.34	1.098	3.2	2.8	S-1.5
[FA][H_2_PO_4_]	S	13.8 (30.8)	44.8	1350	1.027	1.782	77.81	1.758	2.1	4.3	P-0.6
[FA][H_2_PO_4_]-800		22.0 (30.8)^c^	71.4	442	0.264	5.180	52.57	2.184	6.6	12.9	P-4.8
[FA][HSO_4_]	S	24.1 (30.8)	78.2	304	0.128	2.527	77.80	1.638	4.3	8.0	S-1.9
[FA][CF_3_SO_3_]	S	23.6 (29.2)	80.8	654	0.317	2.594	84.39	1.024	2.7	6.1	S-1.2
[FA][BF_4_]	S	23.6 (32.5)	72.6	19	0.041	2.933	84.34	1.278	2.8	4.3	B-0.1
[FA][NO_3_]	S	8.1 (37.5)	21.6	440	0.188	2.656	78.79	1.831	2.2	3.8	
[FA][Cl]	S	26.8 (45.0)	59.6	62	0.027	3.478	84.66	1.293	2.1	5.8	

a *Bulk state at room temperature*.

b *Values in parentheses are theoretical yield calculated based on the overall carbon content in PSs*.

c *Oven yield/theoretical yield*.

d *Determined by combustion elemental analysis (CHN, wt%)*.

e *Determined by high-resolution XPS (at%)*.

The detailed thermal decomposition behaviors are strongly dependent on the anions. Most of the PSs exhibited a significant weight loss below 500°C accompanied with a slight weight loss step. The first step is probably due to the removal of anions while the latter one ascribed to the denitrogenation, and dehydrogenation during polymerization and condensation. It should be noted that the TGA curve of [FA][NO_3_] is nearly vertical to the X axis in the very narrow temperature range of 150-180 °C, indicating a drastic weight loss of up to 72% caused by very fast kinetics. Therefore, the TGA measurement of [FA][NO_3_] must be carried out with a much smaller heating rate of 1°C min^−1^ than that of other [FA][X] PSs (10°C min^−1^). Otherwise, the instant realize of energy at high heating rate will lead to explosion due to the strong oxidative ability of [NO_3_] and high N content in the protic salt, as observed for energetic ILs (Zhang et al., 2014a). Interestingly, [FA][H_2_PO_4_] exhibits three clear weight-loss steps at 150–250°C, 380–580°C, and 800–920°C, respectively. Even at high temperature of 860 °C, a drastic weight loss up to 20% was still observed (Figure 1).

Carbon materials were obtained by direct carbonization of the [FA][X] salts in a horizontal tube furnace at 1,000°C. In order to investigate the weight loss of TGA curve at high temperature for [FA][H_2_PO_4_], a carbon ([FA][H_2_PO_4_]-800) was also obtained at 800°C for comparison. In consistent with the TGA result, all [FA]-based PSs give rise to carbon residues, with variable yields depending on the anion. As shown in Table 1, the yield for [FA][NO_3_] is as low as 8.1 wt%, while it increased to 26.8wt% when the anion was replaced with [Cl]. The calculated relative (oven/theoretical) yield based on the overall C content in the [FA][X] PSs varied from 44.8 to 80.8%. Interestingly, although [FA][HSO_4_] and [FA][H_2_PO_4_] have the same theoretical yield (30.8%) as expected, the resultant carbon yield of [FA][H_2_PO_4_] (13.8wt%) is one half of that of [FA][HSO_4_] (24.1wt%). Our previous work revealed that, for a given basic amine, PSs pairing with weak-acid-based anions such as [BF_4_] and [Cl] usually exhibit very low or even zero carbon yield, mainly due to the possible backward shift from the PSs to the original acid/base pair caused by the low proton-transfer energy of PSs (Zhang et al., 2014b). The substantial carbon yields for all [FA]-based PSs with various anions could be attributed to the strong polymerizable capability of FA even in the presence of weak acid (Gonzalez et al., 1992). Therefore, it is expected that polymerization of FA rather than the backward shifts from PSs to original acid/base occurred for [FA][X] during pyrolysis, which helps to ensure successful carbonization.

### Structure Analysis of N-Doped Porous Carbons

**Figure 2:**
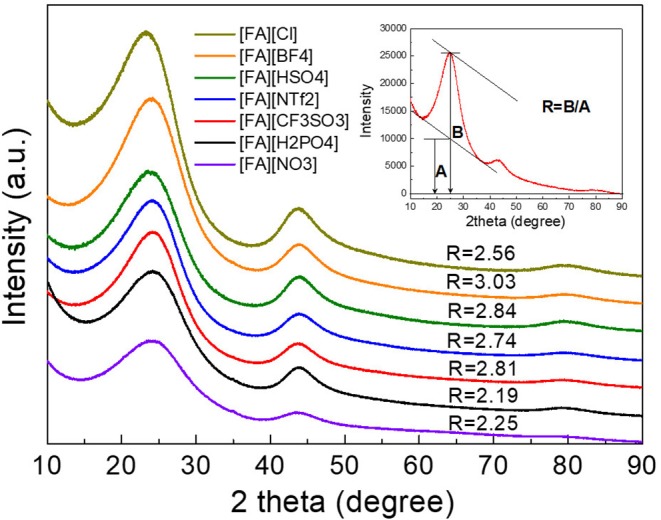
XRD patterns of carbons derived from [FA][X]. The inset is a sketch map for the calculation of the *R*-values.

**Figure 3:**
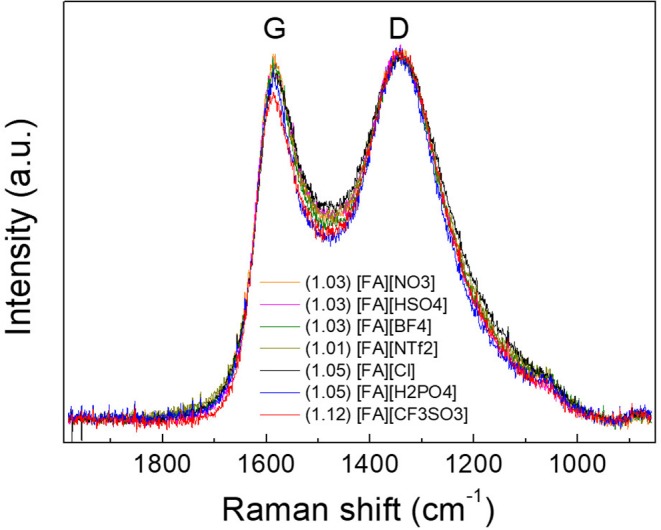
Raman spectra of carbons derived from [FA][X]. The number in the parentheses is the *I*_D_/*I*_G_ ratio.

X-ray diffraction (XRD) results of the resulted carbons are shown in Figure 2. Clearly, two broad diffraction peaks at 24 and 43° corresponding to (002) and (100) diffraction patterns of carbon materials, respectively, were observed for all carbons, which is indicative of the existence of partially nanographitic structures ordinarily found for pyrolytic carbons (Bonino et al., 2005). Based on the established method (Liu et al., 1996; Wang et al., 2009), an empirical parameter (***R***) was used to calculate the average number of carbon nanosheets arranged as single layers. As shown in Figure 2, ***R*** is generally defined as the ratio of height of the (002) peak (B) to the background (A). The general conclusion drawn from previous work is that a larger ***R-***value indicates large number of the parallel single layers in the as-prepared carbon materials and thus a high graphitic degree (Qu, 2008). Depending on the anions in the protic precursors, the ***R***-values of [FA][X]-derived carbons range from 2.19 to 3.03, which are much higher than unit, a value indicating nearly randomly distributed single sheets. Of all samples, carbons derived from [FA][BF_4_], [FA][HSO_4_], [FA][CF_3_SO_3_], and [FA][NTf_2_] (*R* = 3.03, 2.84, 2.81, and 2.74, respectively) exhibit obviously larger ***R*** values than those of other carbons, indicating that these precursors may result in part of edge orientation and decrease the concentration of non-parallel graphene layers (Wang et al., 2009). Figure 3 shows the typical D and G- bands of Raman spectra for all [FA][X]-derived carbons, with little sample-to-sample variations. The broad D band that originates from back scattering of phonon by disorder (edges and defects) in graphene is centered at 1,342 cm^−1^. The location of the G-band ascribed to sp^2^-hybridized graphitic carbon structure is 1,586 cm^−1^, slightly upshifted as compared to 1,582 cm^−1^ observed in graphite (Tuinstra and Koenig, 1970; Nemanich, 1979), which is common for microporous carbons (Tuinstra and Koenig, 1970; Nemanich, 1979; Gonzalez et al., 1992; Liu et al., 1996; Ferrari and Robertson, 2000; Bonino et al., 2005; Qu, 2008; Wang et al., 2009; Martinelli et al., 2011; Zhang et al., 2015d,f). The intensity ratios of D to G-band (I_D_/I_G_) for all samples are above unit, suggesting the presence of many defects in these carbon materials. Among all samples, [FA][CF_3_SO_3_]-derived carbon is more amorphous, as suggested by its high I_D_/I_G_.

**Figure 4:**
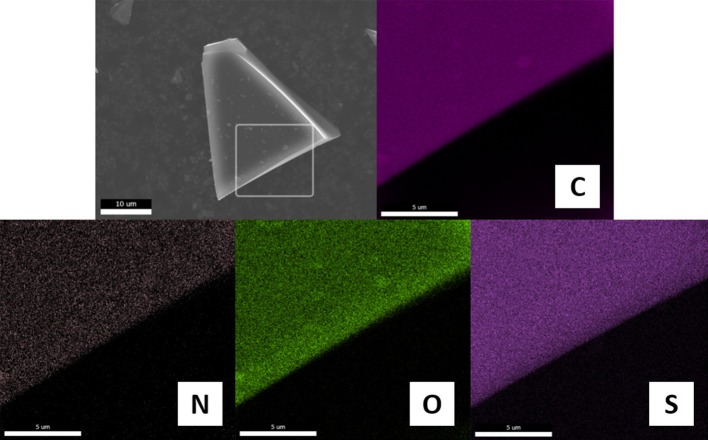
Element distribution from EDS-mapping images of [FA][HSO_4_]-derived carbon. Scale bars in the mapping images are 5 μm.

The relative contents of C, N and H were determined by combustion analysis of carbon materials. As shown in Table 1, all samples are mainly composed of C with contents of higher than 77.8%. In addition, they contain 1.78–3.48% of N with variable content depending on the pairing anions, which are much lower than those of the carbons carbonized directly from PSs containing structure nitrogen such as pyridine or cyano/nitrile groups (Zhang et al., 2014a,b). The pendant alkylamino groups covalently bound to furfuryl moiety are easily removed (e.g., via Hoffmann elimination) due to the weak C-N bonding energy, which may reduce the N contents in the final carbonization products. However, this process may be less affected by the paired anions, because anions are nearly completely released at low-temperature pyrolysis. It should be noted that the total amount of C, N, and H for all carbons is still <90%, which undoubtedly could be due to the presence of other heteroatoms from anions such as O, S, and P. Figure 4 shows the typical elemental distribution of [FA][HSO_4_]-derived carbon. Energy dispersion spectrum (EDS) mapping images demonstrate the presence of C, N, O, and S. These elementals are uniformly dispersed in the whole architecture, reinforced that *in situ* carbonization of a single protic precursor could produce carbon materials with uniform heteroatom dopants. As seen in Supplementary Figure 1, the absence of any other impurities in the EDS analysis of [FA][HSO_4_]-derived carbon make it possible to prepare exclusively metal-free samples.

**Figure 5:**
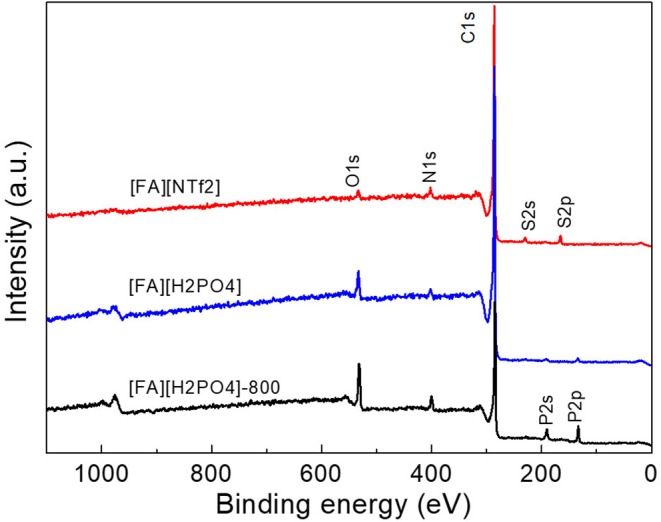
Survey XPS spectra of carbons derived from [FA][X].

The surface elemental composition of carbons was investigated by X-ray photoelectron spectroscopy (XPS). These NPCMs contain mainly C, N, O, and other anion-dependent heteroatoms such as S, P, and B. The total N contents for all carbons fall in the range of 2.1-4.3 at%, while O contents range from 2.8 to 8.0 at% (Table 1). All carbon materials derived from S-containing PSs ([FA][NTf_2_], [FA][HSO_4_], and [FA][CF_3_SO_3_]) are found to yield S-doped NPCMs with S contents of 1.2–1.9 at%. As shown in Figure 5, the survey spectrum of [FA][HSO_4_]-derived carbon demonstrated the presence of C with small amount of N, O, and S inherited from the organic protic salt. It should be noted that O may also originate from the surface absorbed CO_2_ or moisture.

**Figure 6:**
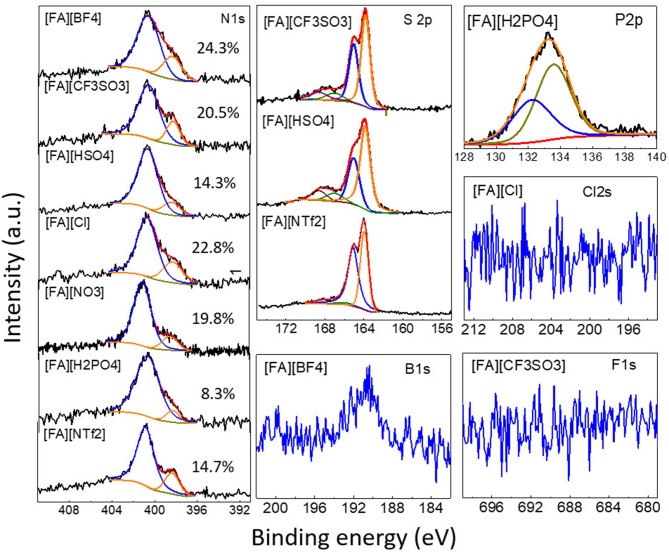
High-resolution N 1s, S 2p, B 1s, P 2p, Cl 2s, and F 1s XPS spectra of [FA][X]-derived carbons. Numbers in the N 1s spectra indicate the percentage of pyridinic N.

In addition, the survey spectra of carbons derived from [FA][H_2_PO_4_] revealed that one can also introduce P into carbon if using [FA][H_2_PO_4_] as the precursor. Clearly, [FA][H_2_PO_4_] carbonized at lower temperature gives rise to abundant heteroatoms (Figure 5). The surface P content of [FA][H_2_PO_4_]-800 determined by high-resolution XPS is 4.8 at%, respectively, much higher than that carbonized at 1,000°C (0.6 at%). Considering the potential applications of P-doped carbon in supercapacitors characteristic of wide operational voltage and large capacitance (Hulicova-Jurcakova et al., 2009), this work is interesting as it provides a simple method to prepare such NPCMs from a single small-molecule precursor. In contrast to N, S and P that are liable to enter the carbon skeleton, other heteroatoms are however difficult to be doped into the final carbon (Table 1 and Figure 6). As seen in Figure 6, signals of F 2s and Cl 1s can hardly be detected by the high-resolution XPS measurement and [FA][BF_4_]-based carbon contains only trace amount of B (<0.1%).

The chemical status of the surface heteroatoms was further investigated by high-resolution XPS spectra. The high-resolution N1s XPS spectra were deconvoluted into Gaussian-Lorenzian shapes to know the details of the chemical bonding. As shown in Figure 6, N1s spectra of all NPCMs can be resolved into two nitrogen species, namely, pyridinic N (398.4 eV) and quaternary N structure (400.8 eV) (Sharifi et al., 2012; Zhang et al., 2015b,c,d,f). Depending on the anion, the percentage of the pyridinic N component varies in the range of 8.3–24.3%. The carbonization temperature also greatly affects the N configuration. For example, with increasing the carbonization temperature of of [FA][H_2_PO_4_] from 800 to 1,000°C, the percentage of pyridinic N in the target NPCMs decreased from 29.8 to 8.3% (Supplementary Figure 2). This is rationalized by the fact that the graphitic N are more thermally stable than the pyridinic N (Tuinstra and Koenig, 1970; Nemanich, 1979; Gonzalez et al., 1992; Pels et al., 1995; Liu et al., 1996; Ferrari and Robertson, 2000; Bonino et al., 2005; Qu, 2008; Hulicova-Jurcakova et al., 2009; Wang et al., 2009; Martinelli et al., 2011; Sharifi et al., 2012; Zhang et al., 2015b,c,f). The S2p spectra can be deconvoluted into several peaks (Figure 6). The two main peaks at binding energies of ~163.9 and 165.0 eV correspond to the S 2p_3/2_ and S 2p_1/2_ of thiophene-like structures, while those at binding energies higher than 166.0 eV was attributed to oxidized sulfur species, as observed for S-doped porous carbons (Yan et al., 2012). Interestingly, the relative percentage of the oxidized sulfur species of [FA][NTf_2_]-derived carbon is significantly lower than that of [FA][HSO_4_] and [FA][CF_3_SO_3_]-derived carbons. The P 2p XPS spectrum of the [FA][H_2_PO_4_]-derived carbon in Figure 6 was comprised of P-O bonding (133.6 eV) and P-C bonding (132.2 eV) (Han et al., 2007; Yang et al., 2012).

**Figure 7:**
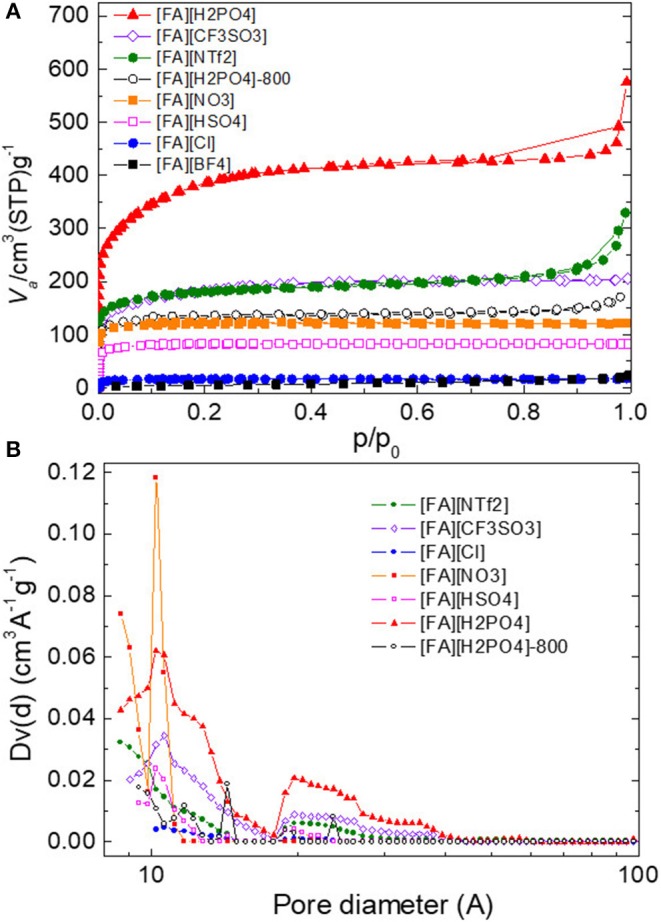
**(A)** Nitrogen sorption isotherms and **(B)** pore size distribution of carbon materials derived from [FA][X].

The porous structure of all NPMCs was examined by nitrogen sorption analysis and was significantly dependent on the anion of the precursors. Figure 7 showed the results of nitrogen adsorption-desorption isotherms and pore size distribution (PSD), and Table 1 gave a summary of the specific surface area (*S*_BET_) and pore volume (*V*_tot_). Generally, bulky anions give rise to large *S*_BET_. For example, carbon derived from [FA][NTf_2_] exhibits a *S*_BET_ as large as 657 m^2^ g^−1^. In contrast, [FA][BF_4_]-based carbon has a much smaller *S*_BET_ of 19 m^2^ g^−1^. This phenomenon is in good agreement with the previous result reported by Dai and co-workers (Lee et al., 2009), and can be explained by the self-template effect of anions during carbonization (Tuinstra and Koenig, 1970; Nemanich, 1979; Gonzalez et al., 1992; Pels et al., 1995; Liu et al., 1996; Ferrari and Robertson, 2000; Bonino et al., 2005; Han et al., 2007; Qu, 2008; Hulicova-Jurcakova et al., 2009; Lee et al., 2009, 2010; Wang et al., 2009; Paraknowitsch et al., 2010; Wang and Dai, 2010; Martinelli et al., 2011; Yang et al., 2011, 2012; Fellinger et al., 2012, 2013; Sharifi et al., 2012; Yan et al., 2012; Fechler et al., 2013; Fulvio et al., 2013; Sahraie et al., 2014; Xie and Su, 2015; Zhang et al., 2015a,b,c,d,f; Zhu et al., 2015). [FA][CF_3_SO_3_] appears to be a more suitable small-molecule carbon precursor because it can yield a porous carbon with high yield of 23.6% and large *S*_BET_ of 654 m^2^ g^−1^ simultaneously. Although [FA][HSO_4_] and [FA][H_2_PO_4_] having anions with same molecular weight and comparable volume, the N_2_ sorption isotherm of [FA][H_2_PO_4_]-derived carbon shows an intensive uptake at low p/p_0_, indicating a gain of the overall surface area. The resultant *S*_BET_ is 1,350 m^2^ g^−1^, much higher than that of [FA][HSO_4_] (304 m^2^ g^−1^). It should be also noted that [FA][H_2_PO_4_] among all [FA][X] samples gave rise to the largest *S*_BET_. This value is comparable to or even higher than that of most highly porous carbon materials generated by templating methods or activation (Supplementary Table 1). In fact, in our previous report, [Aan][HSO_4_] as a precursor can produce porous carbon with *S*_BET_ of 1,380 m^2^/g, while its carbon yield is as low as 6.4% (Zhang et al., 2014b). In contrast, the carbon yield of [FA][H_2_PO_4_] is 13.8%, much higher than that of [Aan][HSO_4_]. Decreasing the carbonization temperature from 1,000 to 800°C was found to dramatically reduce the *S*_BET_ to 442 m^2^ g^−1^, as shown in Table 1. Based on the obvious weight loss in the TGA curve of [FA][H_2_PO_4_], the different P content between 1,000 and 800°C could be contributed by the *in situ* activation effect of [H_2_PO_4_] anion occurred around 850 °C, as did by phosphoric acid for most chemical activation experiments (Puziy et al., 2002a,b). It should be noted that, different from other carbons, both [FA][NTf_2_]- and [FA][H_2_PO_4_]-derived carbons show obvious N_2_ uptakes at high p/p_0_ of 0.9–1.0, which indicates the presence of macropores and large mesopores. The PSD of micropores and small mesopores were calculated by NLDFT method. As shown in Figure 7B, nearly all the carbons contain lots of pores smaller than 4 nm. Most of them have wide PSD, while [FA][NO_3_]-derived carbon displayed a very narrow PSD with a maximum pore size located at 1.0 nm. Among all carbons including [FA][H_2_PO_4_]-800, [FA][H_2_PO_4_] carbonized at 1,000°C gives the most developed microporous and mesoporous structure. The presence of macropores was further confirmed by the FE-SEM images. As seen in Supplementary Figure 3, both [FA][NTf_2_] and [FA][H_2_PO_4_]-derived carbons have rather rough surfaces with structurally opened pores, while all other carbons show very smooth surfaces. HR-TEM images further revealed that all the [FA][H_2_PO_4_]-derived carbons consist of nanoporous structure fabricated by bent graphene layers (Supplementary Figures 4, 5). Particularly, abundant and relatively large nanopores were observed for [FA][H_2_PO_4_]-derived carbon.

### Dye Adsorption

**Figure 8:**
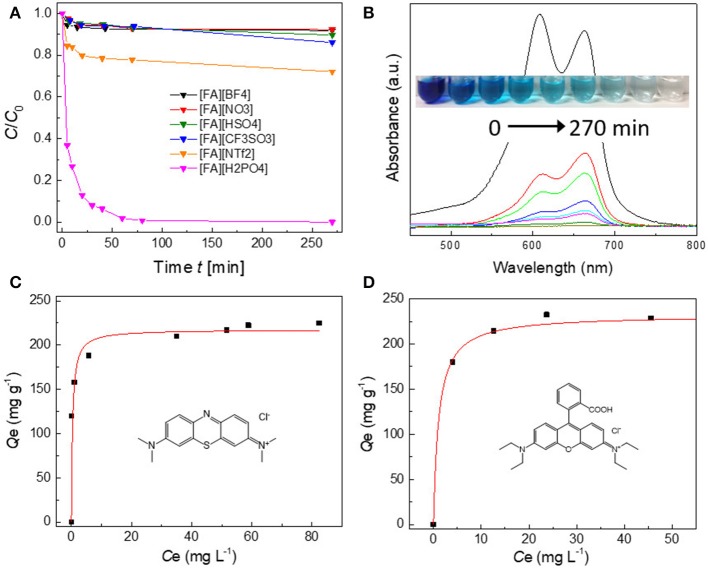
**(A)** Adsorption rates of MB on various porous carbons derived from [FA][X] (80 mg L^−1^, 20 mL, 10 mg carbon). **(B)** UV–vis absorption spectra and corresponding photograph (inset) of the MB aqueous solutions in the presence of [FA][H_2_PO_4_]-derived carbon at different intervals. **(C,D)** Adsorption isotherms of MB and RhB on [FA][H_2_PO_4_]-derived porous carbon, respectively. The insets show the molecular structures of MB and RhB, respectively.

The dye adsorption capability of porous carbons is strongly dependent on the organic precursors. For most [FA][X]-derived carbons, their adsorption is easily saturated within 20 min and <10% can be adsorbed (Figure 8A). In contrast, carbons derived from [FA][NTf_2_] and particularly [FA][H_2_PO_4_] shows improved adsorption capability. [FA][NTf_2_]-derived carbon can adsorb 20% MB within 20 min. The use of [FA][H_2_PO_4_]-derived carbon can remove about 20% of MB from water within 20 min and up to 99% of MB can be adsorbed within 70 min at room temperature. The characteristic band of MB at 663 nm became too weak to be observed after 270 min. At the same time, the aqueous solution becomes completely colorless (inset in Figure 8B), indicating high efficiency for MB removal. Figure 8C demonstrates the equilibrium adsorption isotherms of [FA][H_2_PO_4_]-derived carbon in MB solution. The amount of adsorbed MB drastically increases at very low concentration, reflecting the dye molecule has a high affinity to the carbon surface (Vinu et al., 2006); the adsorption become saturated at a higher equilibrium solution concentration. The type I isotherm can be fitted by the Lanmuir model, which derives a maximum adsorption capacity of 228 mg g^−1^ (Yagub et al., 2014). Compared to other carbon adsorbents (Supplementary Table 2) including porous carbon nanosheets, CNTs, graphene oxide, graphene nanosheet, and activated carbon, [FA][H_2_PO_4_]-derived carbon showed an excellent adsorption performance of MB without any activation. Without question, the adsorption abilities of [FA][X]-derived carbons were determined by the *S*_BET_ of carbons, for which a large *S*_BET_ is expected to give a high capacity. However, it was interestingly found that [FA][NTf_2_]-derived carbon exhibited a much higher capacity than that of [FA][CF_3_SO_3_], despite their comparable *S*_BET_ (657 vs. 654 m^2^ g^−1^). Considering that the PSD for [FA][NTf_2_] and [FA][CF_3_SO_3_]-derived carbons in the region of micropores and small mesopores are close each other (Figure 7B), this result could be ascribed to the presence of macropores and large mesopores in [FA][NTf_2_]-derived carbon, as mentioned above. The micropores and small mespores inside the hierarchical structure could provide high capacity, while the macropores are important for allowing dye molecule diffusion. In this regard, [FA][H_2_PO_4_]-derived carbon, which has the largest *S*_BET_ as well as lots of macropores, gives rise to the highest adsorption capacity as expected. Another cationic dye RhB was also tested to confirm the versatility of this porous carbon. The absorption isotherm of RhB also belongs to type I curve (Langmuir isotherm), with the maximum adsorption capacity of 217 mg g^−1^. [FA][H_2_PO_4_]-derived carbon was superior in terms adsorption capacity for RhB when compared with the values of some reported conventional adsorbents (Supplementary Table 3). In addition, the slightly different adsorption capacities between the two dyes (228 mg g^−1^ for MB vs. 217 mg g^−1^ for RhB) could be probably related to their different molecular size (MB: 0.4 nm × 0.61 nm × 1.43 nm Pelekani and Snoeyink, 2000 vs. RhB: 0.64 nm × 1.09 nm × 1.44 nm) (Huang et al., 2008). As a result, a very small amount of micropores in [FA][H_2_PO_4_]-derived carbon that can adsorb small MB molecules cannot be accessed by large RhB molecules.

## Conclusions

In summary, NPCMs were obtained directly by template-free carbonization of FA-based PSs ([FA][X]). Protonation of FA by various Brønsted acids not only improved the thermal stability, but also facilitated the polymerization, condensation and fusion during carbonization, which finally ensured successful carbonization and yielded NPCMs with anion-dependent structure and properties. Unlike the previous work wherein carbons were generally obtained from only PSs pairing with very few anions, all of the [FA][X] PSs obtained in this study could give rise to substantial carbon yields, from 8.1 wt% for [FA][NO_3_] to 26.8 wt% for [FA][Cl]. The surface areas of the prepared NPCMs varied from 19 to 1,350 m^2^ g^−1^, wherein [FA][H_2_PO_4_]-derived carbon exhibited the largest surface area and pore volume, and highly developed macroporous structure, caused by the *in-situ* activation effect of [H_2_PO_4_] anion during carbonization. These NPCMs contain 1.782–3.478 wt% N and some other heteroatoms such as S and P inherited from the counter anions. Even though F, and Cl are difficult to be doped into the final carbon, this work does provide a very simple method to produce N-doped or N, X-codoped porous carbon materials from a single small-molecule precursor. The obtained NPCMs were further subjected to water treatment by examining their abilities to remove two typical pollutants of organic dyes (MB and RhB). Among all NPCMs, [FA][H_2_PO_4_]-derived carbon exhibited obviously high adsorption capacities toward both MB (228 mg g^−1^) and RhB (217 mg g^−1^) owing to its relatively high surface area as well as developed macropores.

## Data Availability Statement

The raw data supporting the conclusions of this article will be made available by the authors, without undue reservation, to any qualified researcher.

## Author Contributions

YZ directed the work, designed the experiment, synthesized the precursors, analyzed the data, and wrote the manuscript. JW, JD, and SZ prepared and characterized the carbon materials together. GS helped to measure the TEM, analyze the data, and revise the manuscript.

### Conflict of Interest

The authors declare that the research was conducted in the absence of any commercial or financial relationships that could be construed as a potential conflict of interest.
