# Supplementary material for: Template-Free Synthesis of N-Doped Porous Carbon Materials From Furfuryl Amine-Based Protic Salts
Source: Front Chem. 2020 Mar 31;8:196. doi: 10.3389/fchem.2020.00196 (PMC7136577; doi:10.3389/fchem.2020.00196)
Supplement: Supplementary file 1 [file Table_1.DOCX]

Supplementary Material

# Supplementary Figures

#
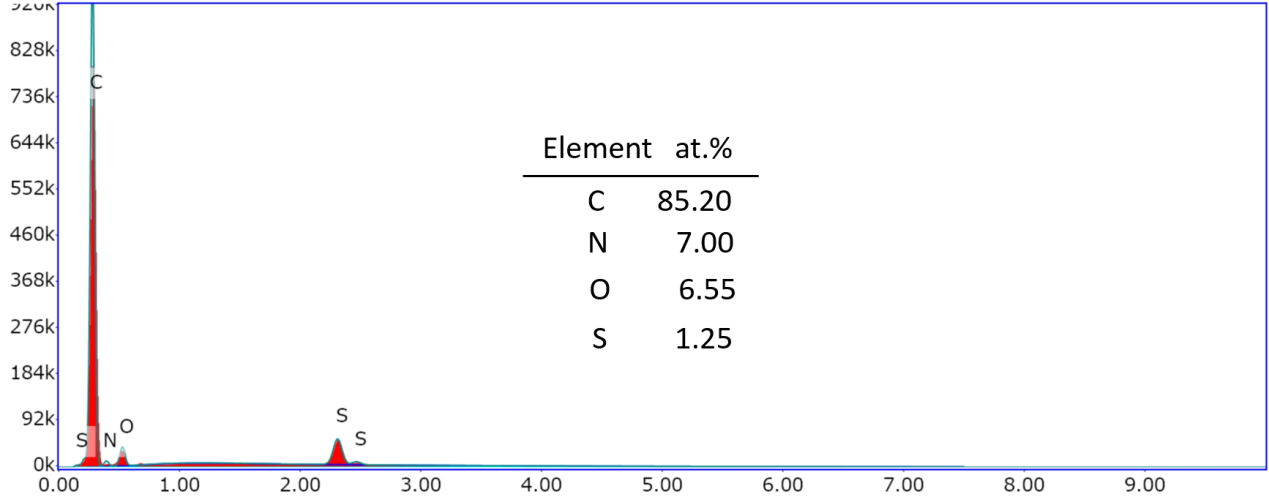
Supplementary Figure 1. Energy dispersive X-ray spectroscopic (EDS) analysis of [FA][HSO4]-derived carbon. Table inside shows respcective elemental content (at.%).


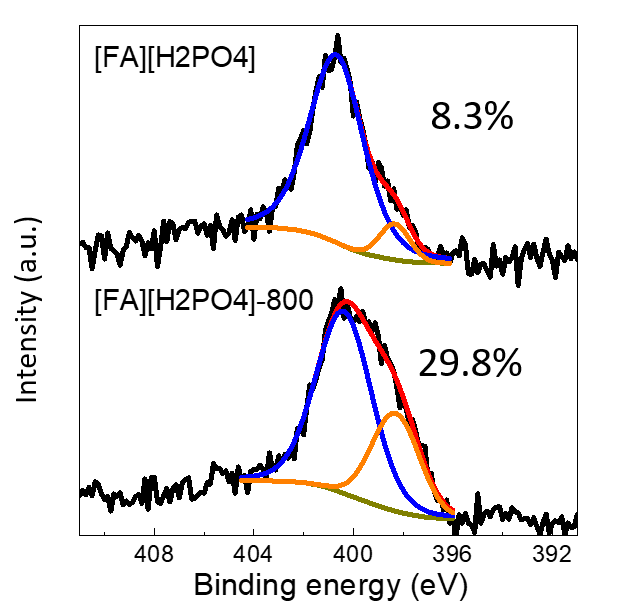


**Supplementary Figure 2.** High-resolution N 1s XPS spectra of [FA][H2PO4]-derived carbons.


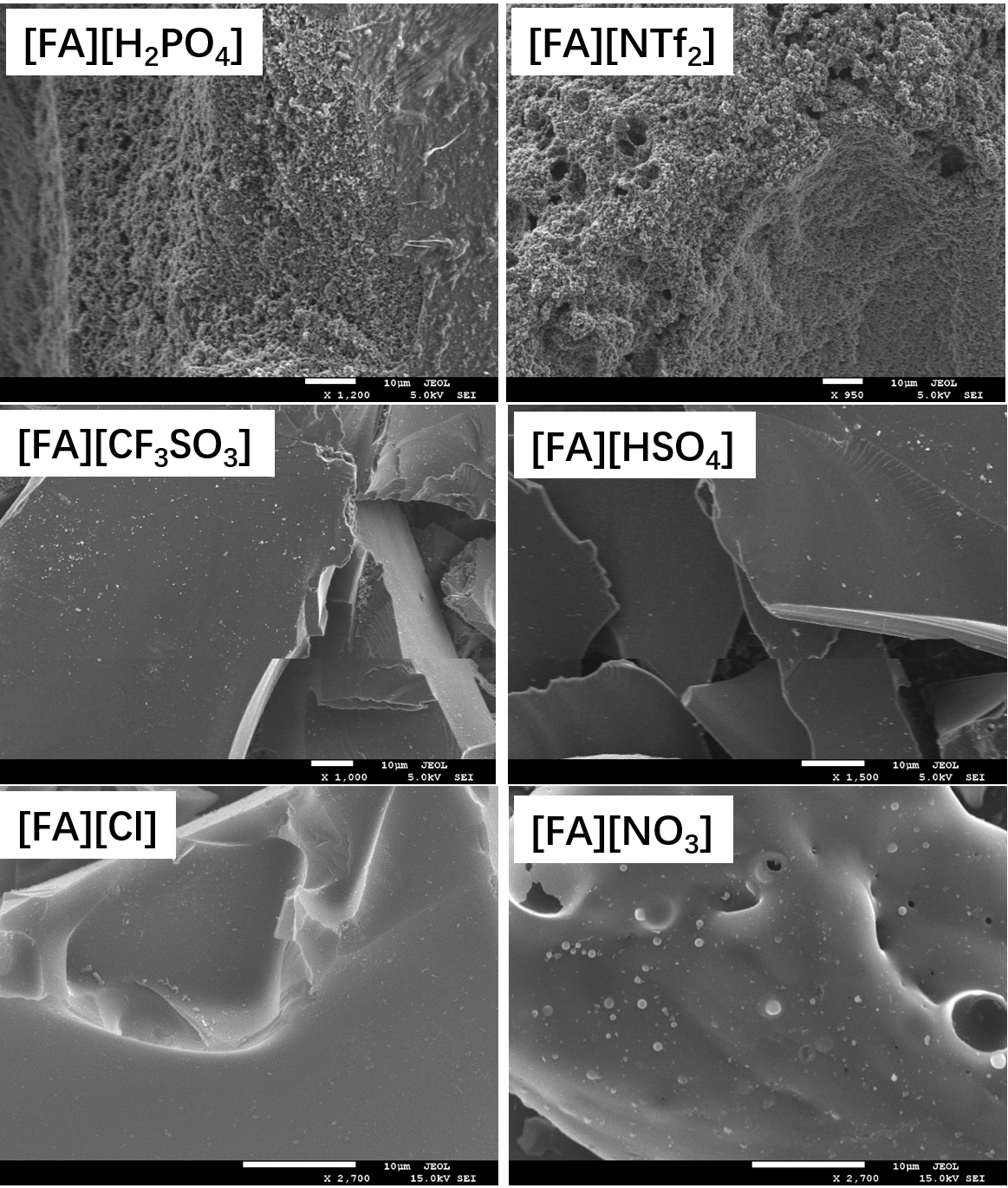


**Supplementary Figure 3.** FE-SEM images of carbons derived from [FA][X].


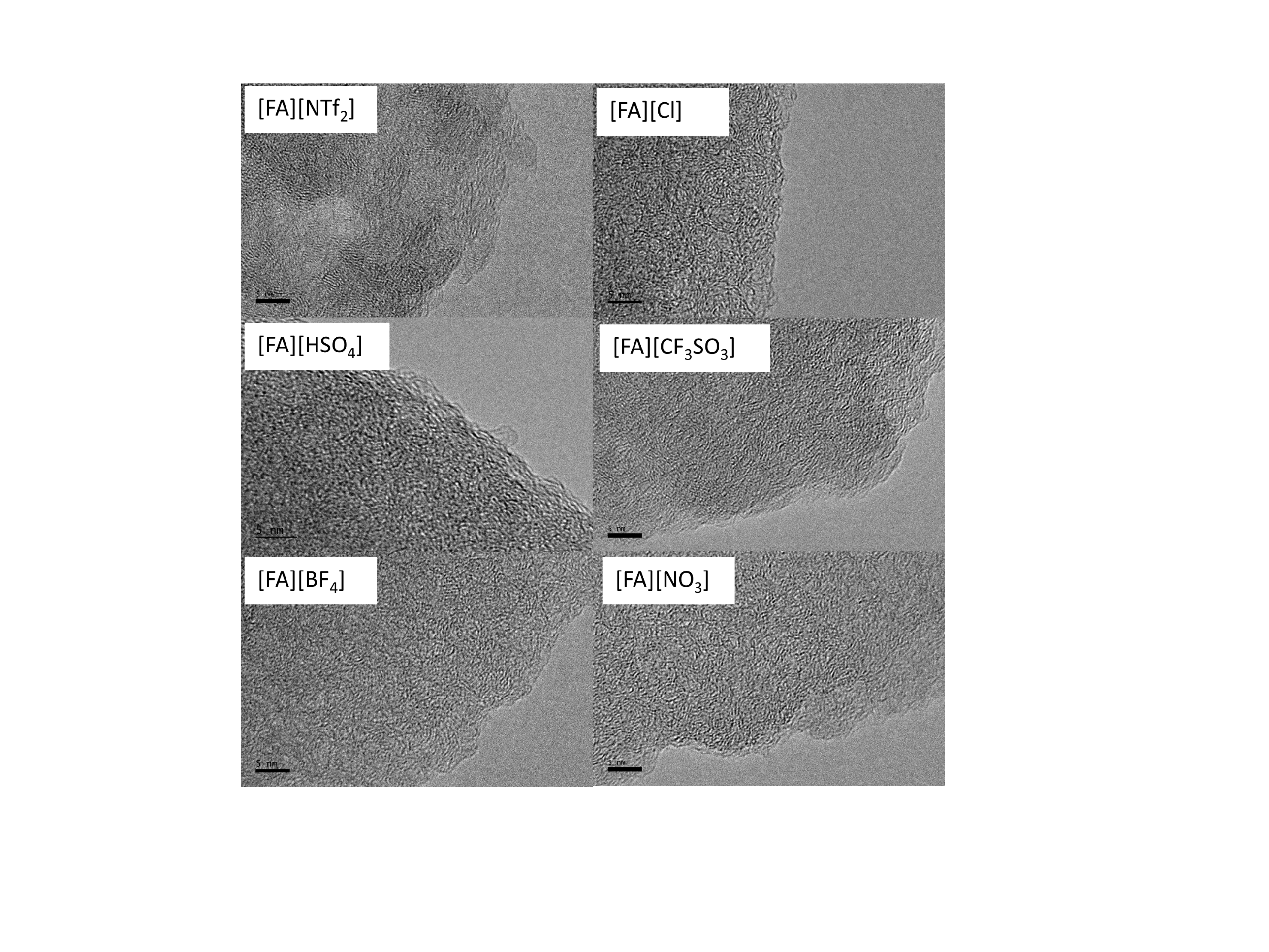


**Supplementary Figure 4.** HR-TEM images of carbons derived from [FA][X].


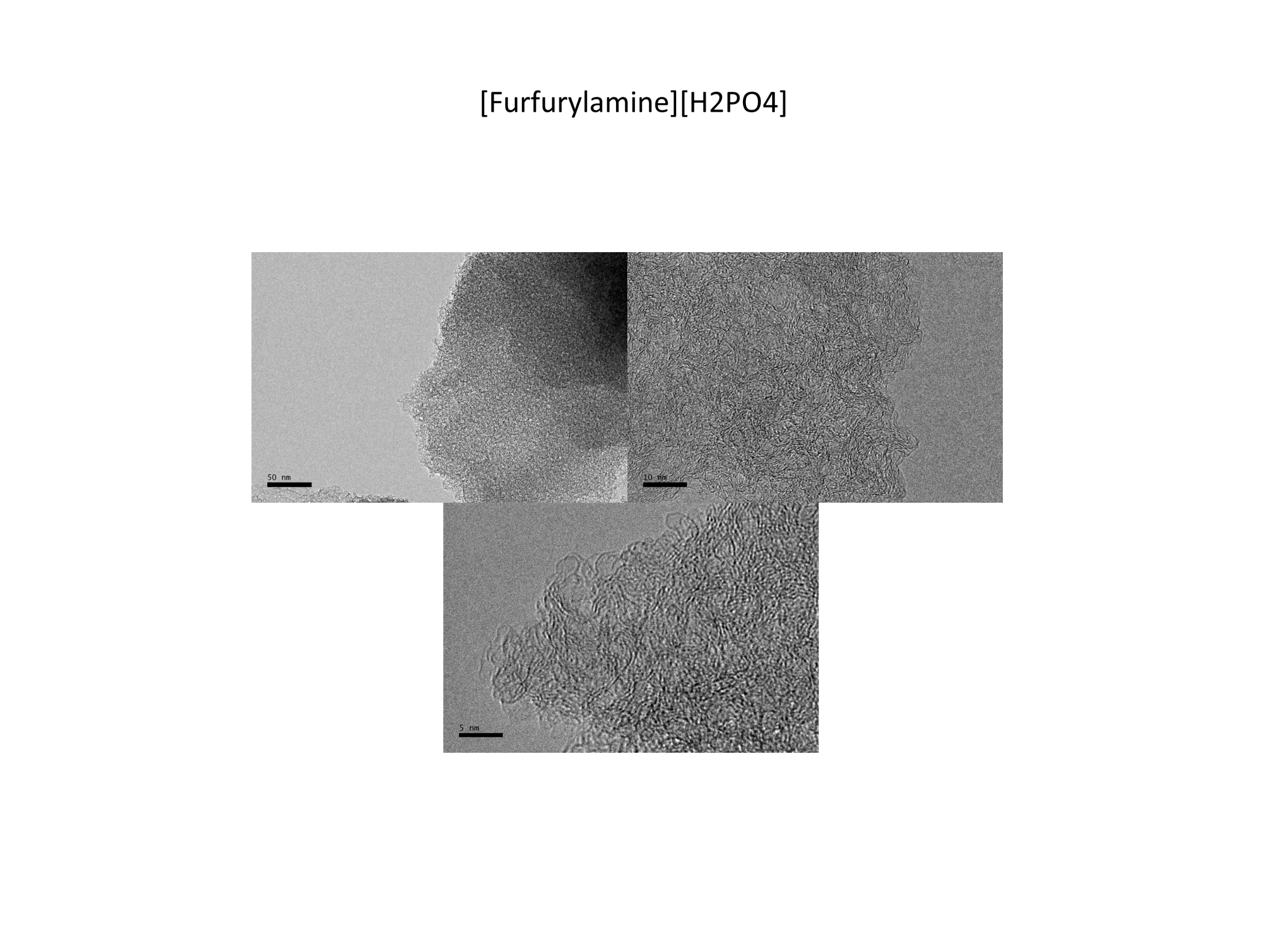


**Supplementary Figure 5.** TEM and HR-TEM images of carbon derived from [FA][H_2_PO_4_].

**Supplementary Table 1** Comparison of the surface area of some highly porous carbon materials.

| Carbon | Preparation method | Surface area (m^2^ g^-1^) | Reference |
| --- | --- | --- | --- |
| KPS-2 | KOH activation | 1337 | J Colloid Interf. Sci. 2002, 250, 93–98 |
| C2-700 | KOH activation | 901 | Carbon 46 (2008) 1159-1174 |
| [Phen][2HSO4]- derived carbon | LUDOX HS-40 as a hard template | 1161 | ChemSusChem, 2015, 8, 1608–1617 |
| EMIM-dca-derived carbon | SBA-15 as a hard template | 906 | Adv. Mater. 2010, 22, 87–92 |
| [Aan][HSO_4_]-derived carbon | Template-free and one-step carbonization | 1380 | J. Am. Chem. Soc., 2014, 136, 1690–1693 |
| [FA][H_2_PO_4_]-derived carbon | Template-free and one-step carbonization | 1350 | This work |

**Supplementary Table 2** Comparison of the adsorption capacity of MB using various carbon adsorbents.

| Adsorbents | Adsorption capacity (mg g^-1^) | Reference |
| --- | --- | --- |
| Porous carbon nanosheets | 30.3 | J. Mater. Chem. A, 2015, 3, 341 |
| CNTs | 35.4–64.7 | Bioresour. Technol., 2010, 101, 3040 |
| Graphene oxide | 64.2 | Chem. Eng. J., 2013, 226, 189 |
| Graphene nanosheet | 111.6 | ACS Nano, 2011, 5, 191 |
| Hollow carbon shell | 238.6 | RSC Adv., 2015, 5, 105047-105056 |
| Activated carbon | 452.2 | J. Hazard. Mater., 2007, 141, 819-825 |
| [FA][H_2_PO_4_]-derived carbon | 228.0 | This work |

**Supplementary Table 3** Comparison of the adsorption capacity of MB using various carbon adsorbents.

| Adsorbents | Adsorption capacity (mg g^-1^) | Reference |
| --- | --- | --- |
| Fe3O4/ bentonite | 62.2 | Appl. Surf. Sci., 2015, 349, 988-996 |
| Tannic acid functionalized graphene | 201 | Colloids Surf., A, 2015, 477, 35-41 |
| Activated carbon prepared from bagasse pith | 98.2 | J. Hazard. Mater., 2008, 168, 1070-1081 |
| Natural zeolite | 37.8 | J. Hazard. Mater., 2006, 136, 946-952 |
| CZIF-867 | 116.2 | RSC Adv., 2015, 5, 105047-105056 |
| Activated carbon | 452.2 | Chem. Eng. J., 2018, 347, 640-647 |
| [FA][H_2_PO_4_]-derived carbon | 217.0 | This work |
